# Development and validation of a prognostic nomogram for early-onset colon cancer

**DOI:** 10.1042/BSR20181781

**Published:** 2019-06-20

**Authors:** Chaoran Yu, Yujie Zhang

**Affiliations:** 1 Fudan University Shanghai Cancer Center, Fudan University, Shanghai 200025, P.R. China; 2 Department of Oncology, Shanghai Medical College, Fudan University, Shanghai 200025, P.R. China; 3 Department of Gastrointestinal Surgery, Shanghai Minimally Invasive Surgery Center, Ruijin Hospital, School of Medicine, Shanghai Jiao Tong University, Shanghai 200025, P.R. China; 4 Department of Gastrointestinal Surgery, Tongji Hospital, Tongji Medical College, Huazhong University of Science and Technology, Wuhan, 430030, P.R. China

**Keywords:** Colon cancer, Cancer-specific survival, Nomogram, Overall survival

## Abstract

The present study was to develop a prognostic nomogram to predict overall survival (OS) and cancer-specific survival (CSS) in early-onset colon cancer (COCA, age < 50). Patients diagnosed as COCA between 2004 and 2015 were retrieved from the surveillance, epidemiology, and end results (SEER) database. All included patients were assigned into training and validation sets. Univariate and multivariate analysis were used to identify independent prognostic variables for the construction of nomogram. The discrimination and calibration plots were used to measure the accuracy of the nomogram. A total of 11220 patients were included from the SEER database. The nomograms were established based on the variables significantly associated with OS and CSS using cox regression models. Calibration plots indicated that both nomograms in OS and CSS exhibited high correlation to actual observed results. The nomograms also displayed improved discrimination power than tumor-node-metastasis (TNM) stage and SEER stage both in the training and validation sets. The monograms established in the present study provided an alternative tool to both OS and CSS prognostic prediction compared with TNM and SEER stages.

## Introduction

Colorectal cancer (CRC) is one of the common malignant death-caused diseases worldwide [1]. In the United States, CRC patients were newly registered in approximately 130000 cases with over 50000 death reports [1]. In Europe, CRC is both the second common cause of death in the European Union with 215000 cases and second common cancer sites with 447000 cases [2]. In Singapore, CRC ranks top in incidence and second in cause of cancer death [3]. Meanwhile, the incidence and death rates of CRC have been increasing in China [4]. Although the incidence and death rates have been reduced in CRC patients older than 50, the incidence of early-onset CRC (age < 50) increases by 22% and the death rate increases by 13% in the United States during the last decade [1].

Radical surgical intervention remains the primary treatment for CRC [5]. Nevertheless, approximately 25% of CRC patients develop recurrence or distant metastasis [6]. Intriguingly, combinational therapies of chemotherapy and targeted drugs have significantly improved the therapeutic benefits in CRC [7,8]. However, the intrinsic complexity of early-onset CRC remains largely unknown. Generally, early-onset tumors are more likely to be associated with germline genotypes. Hong et al. discovered seven genes (CYR61, UCHL1, FOS, FOSB, EGR1, VIP, and KRT24) as a susceptibility gene set associated with early-onset CRC patients [9]. Ågesen et al. indicated that CLC and IFNAR1 were differentially expressed between young and elderly CRC patients with respect to somatic gene expression, highlighting the genomic complexity associated with age [10]. Nevertheless, the overall prognosis of early-onset CRC remains largely constrained by clinical heterogeneity. Therefore, a refined nomogram system is needed to contribute to the prognosis evaluation of early-onset CRC. In fact, given the genomic-features and clinic-management variances between the colon cancer (COCA) and rectal cancer [11,12], the present study exclusively focused on COCA.

Previously, the tumor-node-metastasis (TNM) cancer staging system of American Joint Committee on Cancer (AJCC) has been periodically updated for effective cancer management [13]. However, increasing studies indicated that other factors, including age, race, and tumor site have also been in association with tumor prognosis in individual case [10,14,15]. Therefore, it is needed to establish a prognostic indicator system specified for early-onset COCA patients.

The nomogram-based statistical method has been widely implemented in prognosis-associated clinical studies with comparable results [16,17]. In fact, nomograms enable specifically individual survival scores by dynamically incorporating clinical variables with technical feasibility and reproducibility. However, nomograms for the prognosis of early-onset COCA have not been fully characterized.

Because of this need, a prognostic nomogram based on the large population of COCA data retrieved from the surveillance, epidemiology, and end results (SEER) program (2004–2015) was developed to predict individualized survival in early-onset COCA.

## Materials and methods

### Patients

The clinicopathological data of all COCA patients were retrieved from the SEER program of the United States National Cancer Institute (NCI). SEER program is established to comprehensively collect clinical information on various cancer types for associated incidence, prevalence, and prognostic studies [18,19].

Patients diagnosed with COCA from 2004 to 2015 were retrieved. The histological code [International Classification of Diseases for Oncology, Third edition (ICD-O-3)] and the cancer staging scheme (version 0204) were used to identify all the patients. The inclusion criteria were (1) tumor site was colon excluding rectum; (2) diagnosis had been microscopically confirmed; (3) age <50; (4) complete TNM stage information; (5) COCA was the first and the only cancer primary; (6) surgery had been performed. All the included patients were randomly assigned to a training set and validation set. The present study was performed with the data from SEER program and reference number 14622-Nov2017. No informed consent or institutional review board approval was required in the present study due to the public-available data of SEER.

### Variables

Clinical variables of COCA patients were extracted, including age, sex, marital status, histological grade, tumor site, TNM stage, tumor size, SEER stage follow-up information, and corresponding death causes. Overall survival (OS) was the primary endpoint, defined as the time period from the diagnosis to the death or last follow-up. Cancer-specific survival (CSS) was the second endpoint, defined as the time period from the diagnosis to the death caused by COCA or censoring. Age and tumor size were transformed into categorical variables.

### Construction of the nomogram

All the categorical variables were presented with frequencies and proportions, and analyzed by a chi-square test. The Kaplan–Meier method and log-rank test were used to analyze each potential prognostic variable. All significant variables from univariate analysis were subject to a multivariate Cox proportional hazards analysis. The construction of nomogram was based on the multivariate cox regression model by the R statistical package rms (R Foundation for Statistical Computing, Vienna, Austria).

### Validation of the nomogram

The validation set was used for the validation of the nomogram by the discrimination, calibration, and bootstrap resampling. The concordance index (C-index) was used to measure the difference between the observed and predicted results from the constructed nomogram. Receiver operating characteristics curve (ROC) analysis was performed for sensitivity and specificity. We further compared the nomogram, the TNM stage, and the SEER stage using the C-index. Calibration plot was used to visualize the variance between nomogram-predicted prognosis and actual prognosis. The 45-degree line in a calibration plot was used as a perfect model to compare the actual outcomes. Furthermore, decision curve analysis (DCA) was used to depict the threshold probabilities ranges in comparison with TNM stage and SEER stage. All the statistical analyses were performed using R software version 3.3.0 (Vienna, Austria; www.r-project.org). *P-*value <0.05 was considered as statistically significant.

## Results

### Patient characteristics

**Figure 1:**
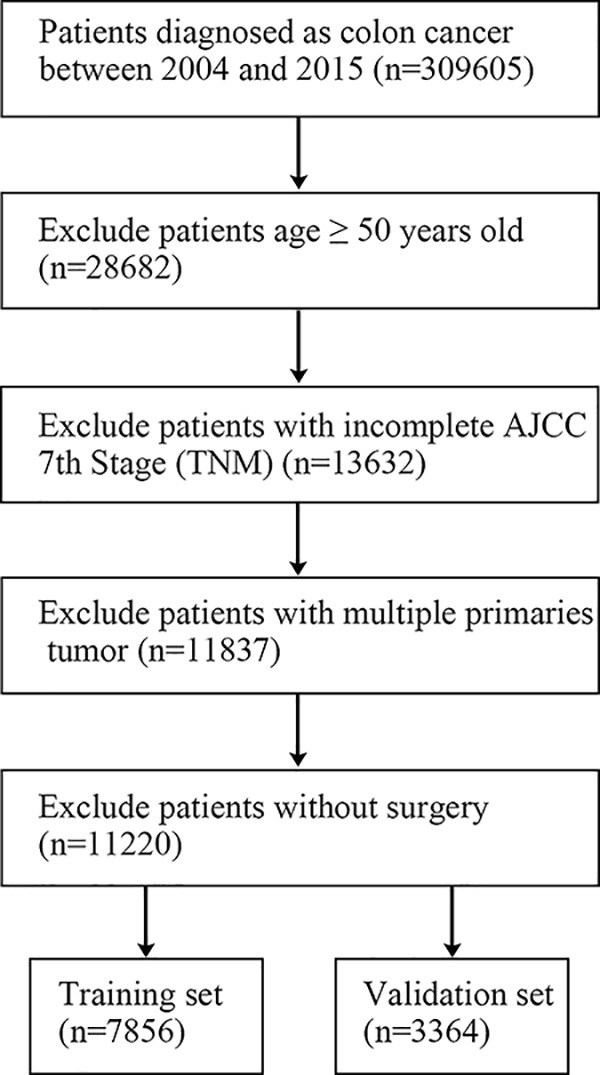
Flow diagram of the included colon cancer patients

A total of 11220 eligible cases were included in the present study with 7856 cases randomly assigned to the training set and 3364 into the validation set (Figure 1). 52.2% of all patients were married and 41.7% were unmarried (also including widowed, single, and divorced). For all the early-onset patients, 7780 were between 40 and 50 years old (69.3%), whereas 2383 were between 30 and 40 years old (21.2%). 1057 were younger than 30 years old (9.4%). The majority of race was white (73.1%). The most common tumor site for COCA in the present study was the sigmoid colon (35.3%), followed by appendix (14.7%) and cecum (13.8%) and ascending colon (12.4%). For tumor size, 2–5 cm was the most common type (38.7%), followed by 5–10 cm (30.7%). 52.5% of all the patients were N0 whereas 80.1% were M0 in AJCC stage system. 42.5% of all the patients were regional in SEER stage (Table 1).

**Table 1:** The demographics and pathological characteristics of included patients

Variables	All patients	Training set	Validation set	*P*-value
	*n* (%)	*n* (%)	*n* (%)	
Total	11220 (100)	7856 (70)	3364 (30)	
Marital status				0.236
Married	5862 (52.2)	4064 (51.7)	1798 (53.4)	
Unmarried	4679 (41.7)	3315 (42.2)	1364 (40.5)	
Unknown	679 (6.1)	477 (6.1)	202 (6.0)	
Sex				0.172
Male	5552 (49.5)	3921 (49.9)	1631 (48.5)	
Female	5668 (50.5)	3935 (50.1)	1733 (51.5)	
Age				0.307
<30	1057 (9.4)	751 (9.6)	306 (9.1)	
30–40	2383 (21.2)	1692 (21.5)	691 (20.5)	
>40	7780 (69.3)	5413 (68.9)	2367 (70.4)	
Race				0.021
White	8206 (73.1)	5700 (72.6)	2506 (74.5)	
Black	1776 (15.8)	1297 (16.5)	479 (14.2)	
Other	1101 (9.8)	768 (9.8)	333 (9.9)	
Unknown	137 (1.2)	91 (1.2)	46 (1.4)	
Grade				0.505
I	1595 (14.2)	1111 (14.1)	484 (14.4)	
II	6658 (59.3)	4654 (59.2)	2004 (59.6)	
III	1720 (15.3)	1233 (15.7)	487 (14.5)	
IV	419 (3.7)	287 (3.7)	132 (3.9)	
Unknown	828 (7.4)	571 (7.3)	257 (7.6)	
Site				0.553
Appendix	1645 (14.7)	1161 (14.8)	484 (14.4)	
Ascending colon	1390 (12.4)	950 (12.1)	440 (13.1)	
Cecum	1552 (13.8)	1115 (14.2)	437 (13.0)	
Descending colon	925 (8.2)	651 (8.3)	274 (8.1)	
Hepatic flexure	364 (3.2)	247 (3.1)	117 (3.5)	
Large intestine, NOS	199 (1.8)	133 (1.7)	66 (2.0)	
Sigmoid colon	3959 (35.3)	2772 (35.3)	1187 (35.3)	
Splenic flexure	359 (3.2)	255 (3.2)	104 (3.1)	
Transverse colon	827 (7.4)	572 (7.3)	255 (7.6)	
AJCC stage				0.96
I	2761 (24.6)	1926 (24.5)	835 (24.8)	
II	2681 (23.9)	1877 (23.9)	804 (23.9)	
III	3547 (31.6)	2495 (31.8)	1052 (31.3)	
IV	2231 (19.9)	1558 (19.8)	673 (20.0)	
AJCC T				0.725
T1	2346 (20.9)	1644 (20.9)	702 (20.9)	
T2	1016 (9.1)	696 (8.9)	320 (9.5)	
T3	5313 (47.4)	3724 (47.4)	1589 (47.2)	
T4	2545 (22.7)	1792 (22.8)	753 (22.4)	
AJCC N				0.548
N0	5891 (52.5)	4117 (52.4)	1774 (52.7)	
N1	3001 (26.7)	2123 (27.0)	878 (26.1)	
N2	2328 (20.7)	1616 (20.6)	712 (21.2)	
AJCC M				0.853
M0	8989 (80.1)	6298 (80.2)	2691 (80.0)	
M1	2231 (19.9)	1558 (19.8)	673 (20.0)	
Tumor size				
≤2cm	1912 (17.0)	1337 (17.0)	575 (17.1)	0.249
>2 to ≤5 cm	4343 (38.7)	3023 (38.5)	1320 (39.2)	
>5 to ≤10 cm	3447 (30.7)	2452 (31.2)	995 (29.6)	
>10 cm	534 (4.8)	360 (4.6)	174 (5.2)	
NA	984 (8.8)	684 (8.7)	300 (8.9)	
SEER stage				0.439
Localized	4054 (36.1)	2809 (35.8)	1245 (37.0)	
Regional	4764 (42.5)	3359 (42.8)	1405 (41.8)	
Distant	2402 (21.4)	1688 (21.5)	714 (21.2)	

### Establishment of the nomogram

**Figure 2:**
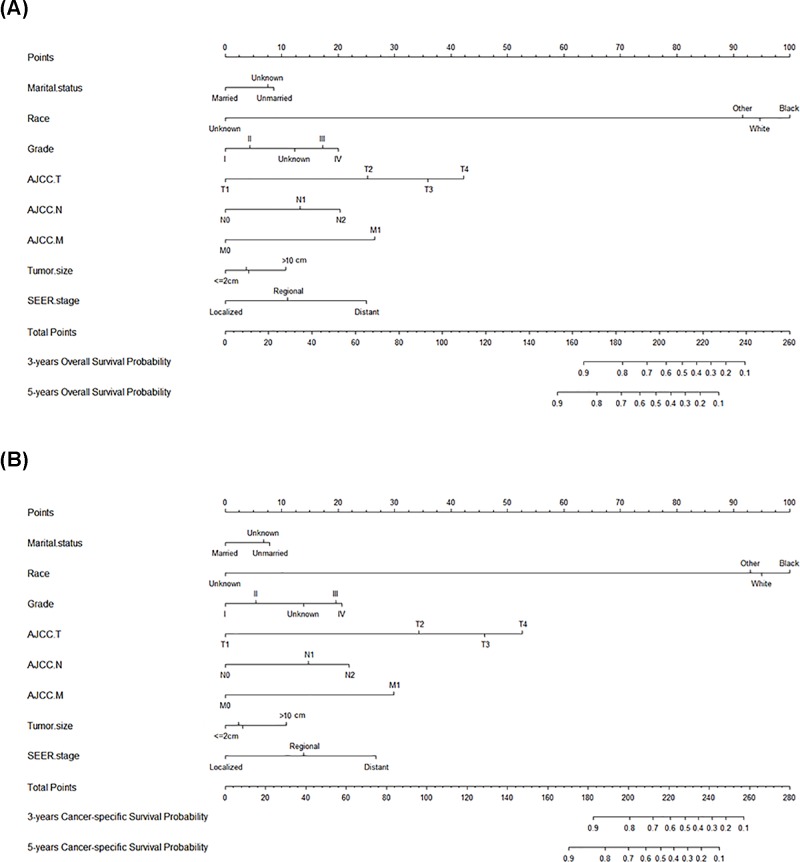
Nomograms for early-onset colon cancer patients (**A**) Nomograms for 3- and 5-year-associated OS. (**B**) Nomograms for 3- and 5-year-associated CSS.

Marital status, age, race, grade, site, TNM stage, tumor size, and SEER stage were significantly associated with OS by univariate analysis in the training set (Table 2). Further multivariate analysis indicated that marital status, race, grade, TNM stage, tumor size, and SEER stage were significantly associated with OS. Therefore, a nomogram of 3- and 5-year OS was established with the independent variables (Figure 2A). In addition, the prognostic values and clinicopathological characterization of patients with different marital status were displayed (Supplementary Figure S1 and Table S1). Moreover, each variable was also examined for CSS and therefore used to build a CSS nomogram as well (Table 3 and Figure 2B).

**Table 2:** Univariate and multivariate analyses of overall survival (OS) in the training set of early-onset colon cancer patients

Variables	Univariate analysis	Multivariate analysis	
	*P-*value	HR(95%CI)	*P-*value
Marital status	<0.001		
Married		Reference	
Unmarried		1.45(1.28–1.64)	<0.001
Unknown		1.39(1.06–1.82)	0.017
Sex	0.215		
Male			
Female			
Age	0.027		
<30		Reference	
30–40		0.96(0.73–1.26)	0.769
>40		1.10(0.86–1.41)	0.452
Race	<0.001		
White		Reference	
Black		1.20(1.04–1.39)	0.015
Other		0.91(0.74–1.12)	0.371
Unknown		9.03e−07(0–Inf)	0.982
Grade	<0.001		
I		Reference	
II		1.22(0.91–1.65)	0.187
III		2.02(1.49−2.75)	<0.001
IV		2.22(1.56–3.16)	<0.001
Unknown		1.66(1.10–2.51)	0.016
Site	<0.001		
Appendix		Reference	
Ascending colon		1.23(0.89–1.70)	0.214
Cecum		1.16(0.85–1.59)	0.348
Descending colon		0.98(0.70–1.40)	0.930
Hepatic flexure		1.12(0.73–1.71)	0.603
Large intestine, NOS		1.31(0.82–2.10)	0.256
Sigmoid colon		0.89(0.66–1.21)	0.467
Splenic flexure		1.10(0.74–1.63)	0.639
Transverse colon		1.07(0.75–1.51)	0.716
AJCC stage	<0.001		
I		–	
II		–	
III		–	
IV		–	
AJCC T	<0.001		
T1		Reference	
T2		2.77(1.37–5.63)	0.005
T3		4.21(2.19–8.10)	<0.001
T4		5.54(2.86–10.72)	<0.001
AJCC N	<0.001		
N0		Reference	
N1		1.73(1.41–2.11)	<0.001
N2		2.34(1.91–2.86)	<0.001
AJCC M	<0.001		
M0		Reference	
M1		2.89(1.93–4.31)	<0.001
Tumor size	<0.001		
≤2cm		Reference	
>2 to ≤5 cm		1.15(0.83–1.60)	0.389
>5 to ≤10 cm		1.16(0.84–1.62)	0.368
>10 cm		1.48(1.00–2.20)	0.0497
SEER stage	<0.001		
Localized		Reference	
Regional		1.61(1.13–2.28)	0.008
Distant		3.03(1.80–5.12)	<0.001

**Table 3:** Univariate and multivariate analyses of Cancer-specific survival (CSS) in the training set of early-onset colon cancer patients

Variables	Univariate analysis	Multivariate analysis	
	*P-*value	HR(95%CI)	*P-*value
Marital status	<0.001		
Married		Reference	
Unmarried		1.40(1.23–1.60)	<0.001
Unknown		1.34(1.01–1.78)	0.041
Sex	0.352		
Male			
Female			
Age	0.054		
<30		Reference	
30–40		0.97(0.73–1.29)	0.829
>40		1.09(0.84–1.41)	0.508
Race	<0.001		
White		Reference	
Black		1.19(1.02–1.39)	0.028
Other		0.95(0.78–1.17)	0.652
Unknown		0.90(0–Inf)	0.984
Grade	<0.001		
I		Reference	
II		1.32(0.96–1.82)	0.092
III		2.27(1.64–3.16)	<0.001
IV		2.33(1.60–3.41)	<0.001
Unknown		1.79(1.15–2.78)	0.010
Site	<0.001		
Appendix		Reference	
Ascending colon		1.09(0.78–1.52)	0.613
Cecum		1.01(0.73–1.39)	0.953
Descending colon		0.85(0.59–1.21)	0.367
Hepatic flexure		1.05(0.68–1.61)	0.830
Large intestine, NOS		1.20(0.75–1.94)	0.450
Sigmoid colon		0.80(0.59–1.09)	0.156
Splenic flexure		0.94(0.63–1.42)	0.778
Transverse colon		0.94(0.66–1.34)	0.730
AJCC stage	<0.001		
I		–	
II		–	
III		–	
IV		–	
AJCC T	<0.001		
T1		Reference	
T2		4.12(1.66–10.18)	0.002
T3		6.50(2.77–15.24)	<0.001
T4		8.59(3.64–20.23)	<0.001
AJCC N	<0.001		
N0		Reference	
N1		1.88(1.52–2.32)	<0.001
N2		2.55(2.06–3.16)	<0.001
AJCC M	<0.001		
M0		Reference	
M1		3.35(2.17–5.17)	<0.001
Tumor size	<0.001		
≤2cm		Reference	
>2 to ≤5 cm		1.11(0.79–1.55)	0.565
>5 to ≤10 cm		1.13(0.80–1.59)	0.484
>10 cm		1.51(1.01–2.27)	0.046
SEER stage	<0.001		
Localized		Reference	
Regional		1.79(1.20–2.66)	0.004
Distant		3.16(1.77–5.66)	<0.001

### Nomogram validation

**Figure 3:**
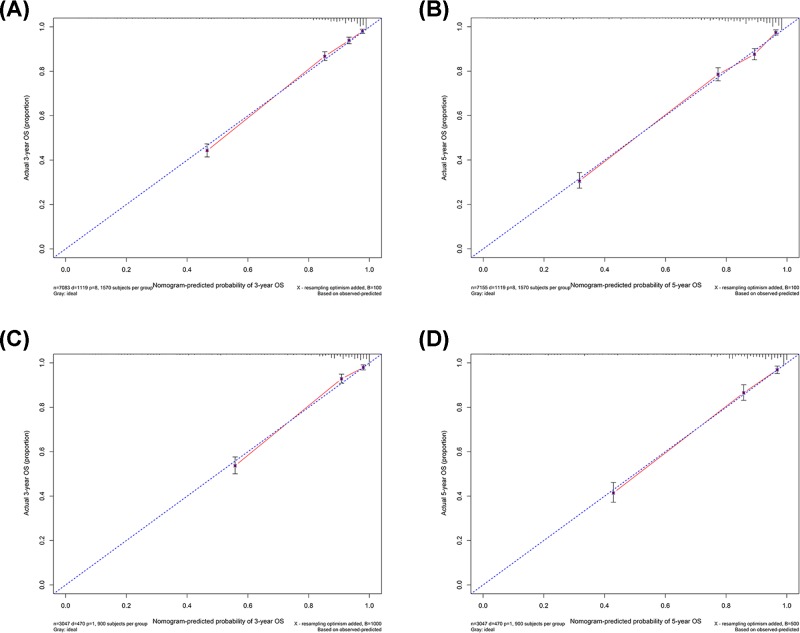
Calibration plots of the training and validation sets for the OS-associated nomograms (**A,B**) The calibration plots of the training set in 3- and 5-year OS. (**C,D**) The calibration plots of the validation set in 3- and 5-year OS.

**Figure 4:**
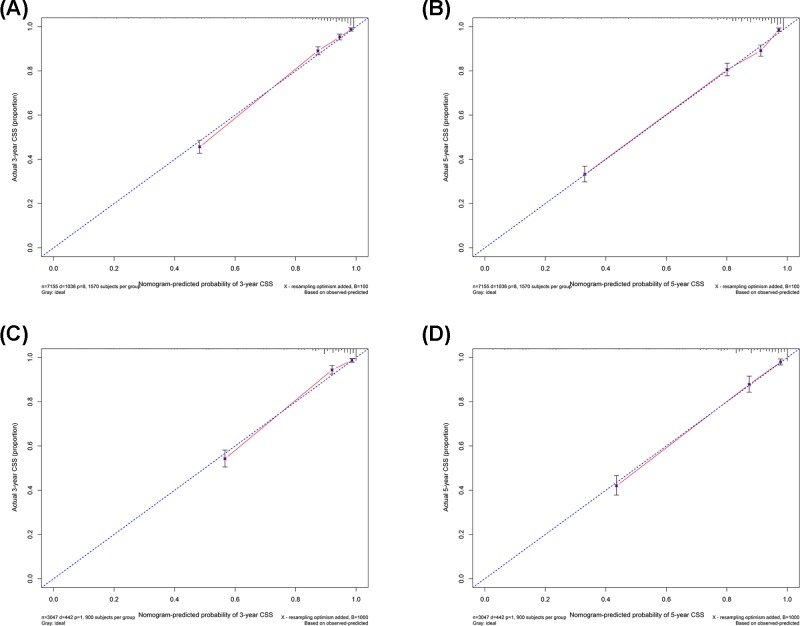
Calibration plots of the training and validation sets for the CSS-associated nomograms (**A,B**) The calibration plots of the training set in 3- and 5-year CSS. (**C,D**) The calibration plots of the validation set in 3- and 5-year CSS.

The nomograms were both internally and externally validated. The internal validation was performed via the training cohort with the C-index as 0.835 (95%CI, 0.823–0.847) in OS and 0.851 (95%CI, 0.840–0.862) in CSS, respectively (Table 4). The external validation was performed via the validation cohort with the C-index as 0.848 (95%CI, 0.831–0.865) in OS and 0.863 (95%CI, 0.847–0.879) in CSS, respectively. Calibration plots of the validations of OS and CSS nomograms indicated highly correlations between the predicted and observed results (Figures 3 and 4).

**Table 4:** Comparison of C-indexes between the nomograms, TNM, and SEER stages in early-onset colon cancer patients

		Training set			Validation set		
		HR	95%CI	*P*-value	HR	95%CI	*P*-value
OS	Nomogram	0.835	0.823–0.847		0.848	0.831–0.865	
	SEER stage	0.780	0.767–0.793	<0.001	0.798	0.780–0.816	<0.001
	TNM 7th stage	0.818	0.806–0.830	0.027	0.84	0.823–0.857	0.126
CSS	Nomogram	0.851	0.840–0.862		0.863	0.847–0.879	
	SEER stage	0.795	0.783–0.807	<0.001	0.813	0.795–0.831	<0.001
	TNM 7th stage	0.835	0.823–0.847	0.034	0.858	0.842–0.874	0.189

**Figure 5:**
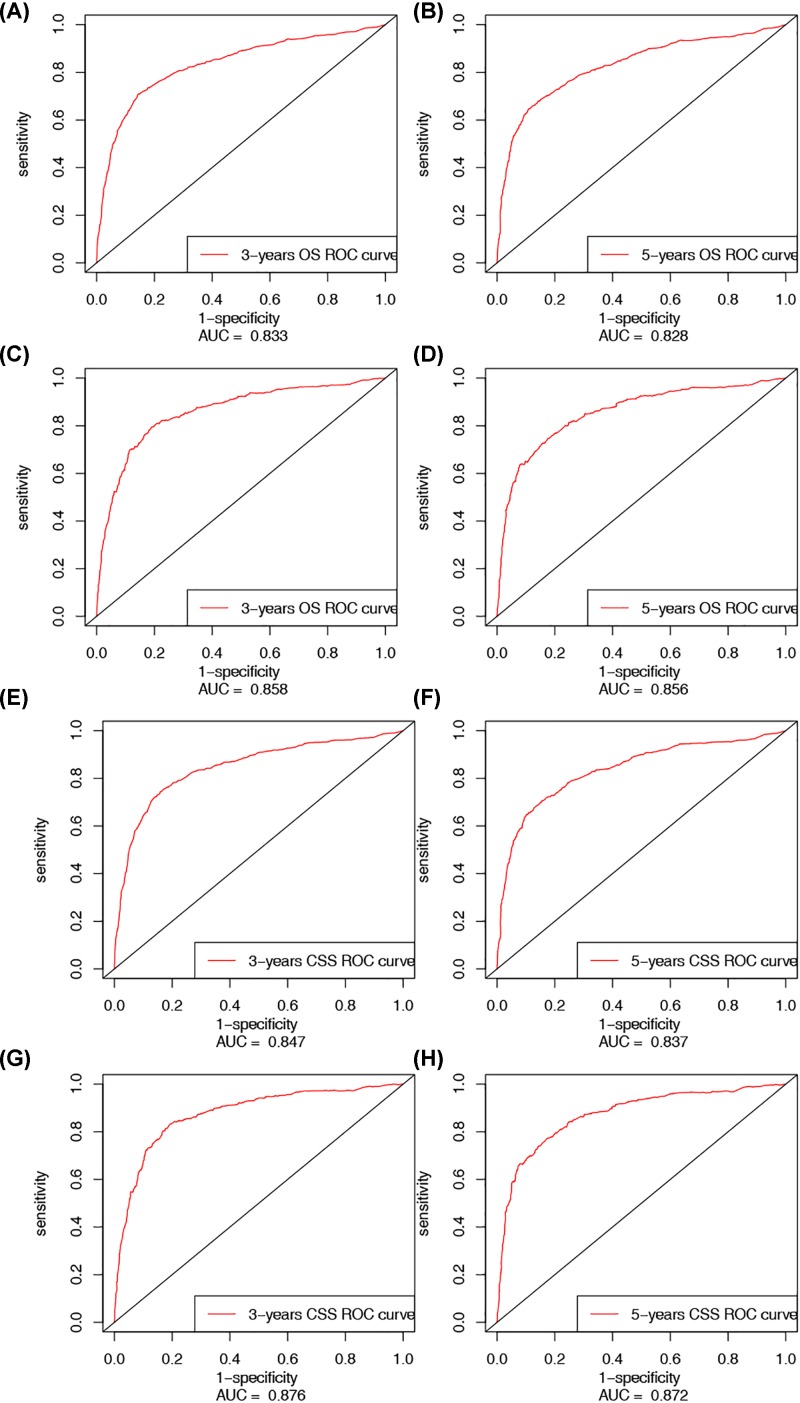
ROC analysis for training and validation sets (**A**) The ROC of 3 years OS for training set; (**B**) the ROC of 5 years OS for training set; (**C**) the ROC of 3 years OS for validation set; (**D**) the ROC of 5 years OS for validation set; (**E**) the ROC of 3 years CSS for training set; (**F**) the ROC of 5 years CSS for training set; (**G**) the ROC of 3 years CSS for validation set; and (**H**) the ROC of 5 years CSS for validation set.

**Figure 6:**
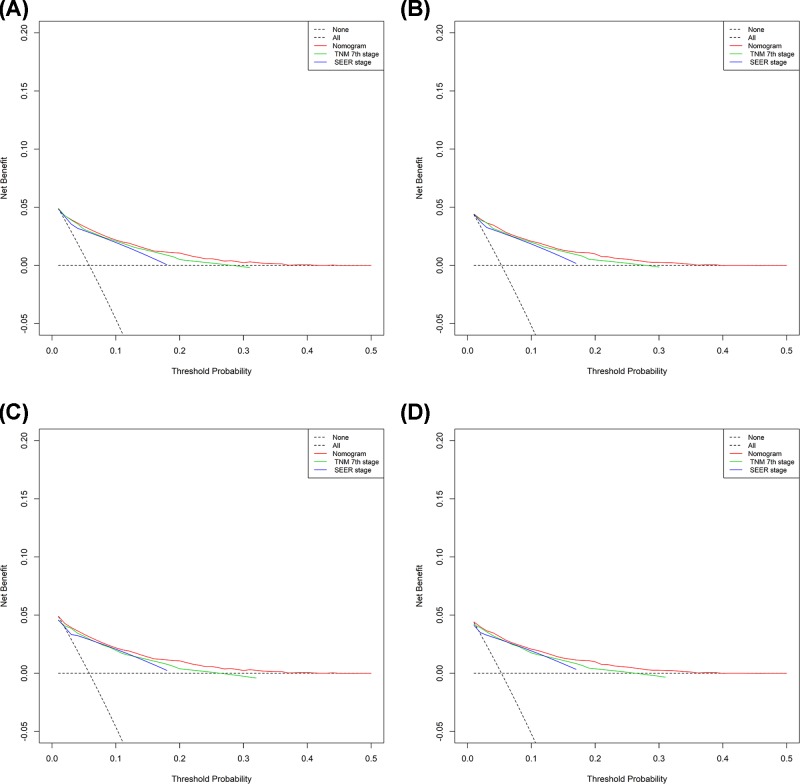
DCA of the training and validation sets for the CSS- and OS-associated nomograms (**A,B**) The DCA of nomogram in training set for OS (A) and CSS (B). (**C,D**) The DCA of nomogram in validation set for OS (C) and CSS (D).

The area under ROC curve (AUC) was analyzed for both the training and validation set, respectively (Figure 5A–H). Furthermore, the comparisons between the nomograms and TNM stage and SEER stage were performed. The OS and CSS nomograms showed comparable results to TNM stage and SEER stage in both training and validation sets (Table 4). Moreover, the DCA results of nomograms also strengthened the clinical applicability of nomograms from OS and CSS with superiority over TNM stage and SEER stage (Figure 6).

## Discussion

The present study established OS and CSS prognostic nomograms for COCA patients derived from the SEER program with favorable discrimination and calibration and comparable predictive capability. In fact, the nomogram highlighted the clinical significance of marital status, race, grade, TNM stage, tumor size, and SEER stage in early-onset COCA patients.

The role of marital status in cancer had been previously investigated in SEER program [20]. Married patients were featured by less metastatic diseases, reduced cancer-specific deaths, and more likely to receive definitive therapy [20]. For colon cancer, married patients were more likely to be diagnosed at earlier stage and to receive surgical treatment [21]. Moreover, married patients had significantly lower risk in CSS [21]. Our study also indicated the consistent results.

Noteworthy, race, sex, and tumor site were not an independent prognostic variable for early-onset COCA patients in the present study. In fact, race and sex has been viewed as one of the essence variables for cancer treatment [22–24]. Tumor site in CRC had been intensively studied with large population [25,26]. Right-sided colon cancers exhibited increased mortality risk compared with left-sided colon cancers. However, no specific tumor site has been under investigation. Intriguingly, although age was associated with significant prognosis in univariate analysis, it remained insignificant in multivariate analysis, indicating that the subtle stratification between age <30, 30–40, and 40–50 required further investigation.

Of note, tumor size was an independent prognostic variable in the nomogram in the present study. In fact, only the tumor >10 cm exhibited significant higher prognostic risk than tumor <2cm whereas the rest stratification remained insignificant. It was possible that the tumor size could be one of the insightful variables for the prognostic risk prediction.

Up to now, nomogram statistical tool provided reasonable, reproducible, and rigid algorithms for individualized prognostic assessment. It has been implemented as prognostic indicators for several cancer types including pancreas, gastrointestinal stromal tumor (GIST), and gastric cancer [16,27,28]. In pancreatic cancer, a nomogram constructed by CSS data of 53028 patients diagnosed as pancreatic cancer from the SEER program and eight independent clinical variables (C-index = 0.734) [16]. For a total of 5622 GIST patients, similar nomograms were also established by seven independent clinical variables in both CSS and OS data. Noteworthy, these nomogram exhibited better discrimination power than TNM stage and SEER stage systems [27]. Furthermore, Liu et al. identified a prognostic nomogram for disease specific survival (DSS) of gastric cancer patients using the SEER program and external validation set [28]. Collectively, incorporation of numerous prognostic-associated clinical variables into nomogram algorithms could display comparable staging system and more disease-specific features.

The limitations of the present study were the lack of external clinical data from independent sources and the vacant data on chemotherapy and radiotherapy, as well as the genomic phenotypes in SEER program. Moreover, given the potential confounding factors within the surgical patterns, surgical styles, postoperative complications, and some surgical items remaining contradictory, the COCA patients without surgery or the complete TNM stage data were excluded from the present study.

## Conclusion

The nomograms established in the present study provided an alternative tool to both OS and CSS prognostic prediction compared with TNM and SEER stages.

## Availability of data and materials

The datasets supporting the conclusion of this article are included within the article.

## Human participants and animal rights

This article does not contain any studies with human participants or animals performed by any of the authors.

## Supporting information

**Supplementary Figure S1:** 

**Supplementary Table S1:** Clinicopathological characterization of early-onset colon cancer patients stratified by marital status.

## Abbreviations

AUC: area under receiver operating characteristics curve
AJCC: American Joint Committee on Cancer
95%CI: 95% confidence intervals
C-index: concordance index
COCA: colon cancer
CRC: colorectal cancer
CSS: cancer-specific survival
DCA: decision curve analysis
GIST: gastrointestinal stromal tumor
HR: hazard ratio
ICD-O-3: International Classification of Diseases for Oncology, Third edition
NCI: National Cancer Institute
OS: overall survival
ROC: receiver operating characteristics curve
SEER: surveillance, epidemiology, and end results
TNM: tumor-node-metastasis stage

## References

[1] Siegel R.L., Miller K.D., Fedewa S.A. (2017) Colorectal cancer statistics, 2017. CA Cancer J. Clin. 67, 177–193 28248415 10.3322/caac.21395

[2] Ferlay J., Steliarova-Foucher E., Lortet-Tieulent J., Rosso S., Coebergh J.W., Comber H. (2013) Cancer incidence and mortality patterns in Europe: estimates for 40 countries in 2012. Eur. J. Cancer 49, 1374–1403 10.1016/j.ejca.2012.12.027 23485231

[3] Chia K.S., Seow A., Lee H.P. and Shanmugaratnam K. (2000) Cancer incidence in Singapore 1993–1997. Singapore cancer registry report

[4] Chen W., Zheng R., Baade P.D. (2016) Cancer statistics in China, 2015. CA Cancer J. Clin. 66, 115–132 26808342 10.3322/caac.21338

[5] Benson A.B., Venook A.P., Cederquist L., Chan E., Chen Y.J., Cooper H.S. (2017) Colon cancer, version 1. 2017, NCCN clinical practice guidelines in oncology. J. Natl. Compr. Cancer Netw. 15, 370–398 10.6004/jnccn.2017.0036 28275037

[6] Van Cutsem E., Nordlinger B., Adam R., Köhne C.H., Pozzo C., Poston G. (2006) Towards a pan-European consensus on the treatment of patients with colorectal liver metastases. Eur. J. Cancer 42, 2212–2221 10.1016/j.ejca.2006.04.012 16904315

[7] Cunningham D., Humblet Y., Siena S., Khayat D., Bleiberg H., Santoro A. (2004) Cetuximab monotherapy and cetuximab plus irinotecan in irinotecan-refractory metastatic colorectal cancer. N. Engl. J. Med. 351, 337–345 10.1056/NEJMoa033025 15269313

[8] Hurwitz H., Fehrenbacher L., Novotny W., Cartwright T., Hainsworth J., Heim W. (2004) Bevacizumab plus irinotecan, fluorouracil, and leucovorin for metastatic colorectal cancer. N. Engl. J. Med. 350, 2335–2342 10.1056/NEJMoa032691 15175435

[9] Hong Y., Ho K.S., Eu K.W. and Cheah P.Y. (2007) A susceptibility gene set for early onset colorectal cancer that integrates diverse signaling pathways: implication for tumorigenesis. Clin. Cancer Res. 13, 1107–1114 10.1158/1078-0432.CCR-06-1633 17317818

[10] Ågesen T.H., Berg M., Clancy T., Thiis-Evensen E., Cekaite L., Lind G.E. (2011) CLC and IFNAR1 are differentially expressed and a global immunity score is distinct between early-and late-onset colorectal cancer. Genes Immun. 12, 653 10.1038/gene.2011.43 21716316

[11] Cancer Genome Atlas Network (2012) Comprehensive molecular characterization of human colon and rectal cancer. Nature 487, 330 10.1038/nature11252 22810696 PMC3401966

[12] Minsky B.D. (2011) Unique considerations in the patient with rectal cancer. In Seminars in Oncology, vol. 38, pp. 542–551, WB Saunders 10.1053/j.seminoncol.2011.05.008 21810513

[13] Edge S.B. and Compton C.C. (2010) The American Joint Committee on Cancer: the 7th edition of the AJCC cancer staging manual and the future of TNM. Ann. Surg. Oncol. 17, 1471–1474 10.1245/s10434-010-0985-4 20180029

[14] Liang L., Zeng J.H., Qin X.G., Chen J.Q., Luo D.Z. and Chen G. (2018) Distinguishable prognostic signatures of left-and right-sided colon cancer: a study based on sequencing data. Cell. Physiol. Biochem. 48, 475–490 10.1159/000491778 30016783

[15] Wang J., Sun Y. and Bertagnolli M.M. (2015) Comparison of gastric cancer survival between Caucasian and Asian patients treated in the United States: results from the Surveillance Epidemiology and End Results (SEER) database. Ann. Surg. Oncol. 22, 2965–2971 10.1245/s10434-015-4388-4 25631065

[16] Song W., Miao D.L. and Chen L. (2018) Nomogram for predicting survival in patients with pancreatic cancer. Onco Targets Therapy 11, 539 10.2147/OTT.S154599 29416354 PMC5790064

[17] He C., Zhang Y., Cai Z., Lin X. and Li S. (2018) Overall survival and cancer-specific survival in patients with surgically resected pancreatic head adenocarcinoma: a competing risk nomogram analysis. J. Cancer 9, 3156 10.7150/jca.25494 30210639 PMC6134825

[18] Hankey B.F., Ries L.A. and Edwards B.K. (1999) The surveillance, epidemiology, and end results program: a national resource. Cancer Epidemiol. Biomarkers Prev. 8, 1117–1121 10613347

[19] Hayat M.J., Howlader N., Reichman M.E. and Edwards B.K. (2007) Cancer statistics, trends, and multiple primary cancer analyses from the Surveillance, Epidemiology, and End Results (SEER) Program. Oncologist 12, 20–37 10.1634/theoncologist.12-1-20 17227898

[20] Aizer A.A., Chen M.H., McCarthy E.P., Mendu M.L., Koo S., Wilhite T.J. (2013) Marital status and survival in patients with cancer. J. Clin. Oncol. 31, 3869 10.1200/JCO.2013.49.6489 24062405 PMC4878087

[21] Wang L., Wilson S.E., Stewart D.B. and Hollenbeak C.S. (2011) Marital status and colon cancer outcomes in US Surveillance, Epidemiology and End Results registries: does marriage affect cancer survival by gender and stage? Cancer Epidemiol. 35, 417–422 10.1016/j.canep.2011.02.004 21466984

[22] Goggins W.B. and Lo F.F. (2012) Racial and ethnic disparities in survival of US children with acute lymphoblastic leukemia: evidence from the SEER database 1988–2008. Cancer Causes Control 23, 737–743 22450738 10.1007/s10552-012-9943-8

[23] Shavers V.L. and Brown M.L. (2002) Racial and ethnic disparities in the receipt of cancer treatment. J. Natl. Cancer Inst. 94, 334–357 10.1093/jnci/94.5.334 11880473

[24] Zeng C., Wen W., Morgans A.K., Pao W., Shu X.O. and Zheng W. (2015) Disparities by race, age, and sex in the improvement of survival for major cancers: results from the National Cancer Institute Surveillance, Epidemiology, and End Results (SEER) Program in the United States, 1990 to 2010. JAMA Oncol. 1, 88–96 10.1001/jamaoncol.2014.161 26182310 PMC4523124

[25] Siegel R.L., Ward E.M. and Jemal A. (2012) Trends in colorectal cancer incidence rates in the United States by tumor location and stage, 1992–2008. Cancer Epidemiol. Biomarkers Prev. 21, 411–416 10.1158/1055-9965.EPI-11-1020 22219318

[26] Meguid R.A., Slidell M.B., Wolfgang C.L., Chang D.C. and Ahuja N. (2008) Is there a difference in survival between right-versus left-sided colon cancers? Ann. Surg. Oncol. 15, 2388 10.1245/s10434-008-0015-y 18622647 PMC3072702

[27] Song W., Lv C.G., Miao D.L., Zhu Z.G., Wu Q., Wang Y.G. (2018) Development and validation of a nomogram for predicting survival in patients with gastrointestinal stromal tumours. Eur. J. Surg. Oncol. 44, 1657–1665 10.1016/j.ejso.2018.07.004 30082175

[28] Liu J., Geng Q., Liu Z., Chen S., Guo J., Kong P. (2016) Development and external validation of a prognostic nomogram for gastric cancer using the national cancer registry. Oncotarget 7, 35853 27016409 10.18632/oncotarget.8221 PMC5094968

