# Supplementary material for: Development and validation of a prognostic nomogram for early-onset colon cancer
Source: Biosci Rep. 2019 Jun 20;39(6):BSR20181781. doi: 10.1042/BSR20181781 (PMC6617053; doi:10.1042/BSR20181781)
Supplement: Supplementary file 1 [file bsr20181781_Supp1.pdf]

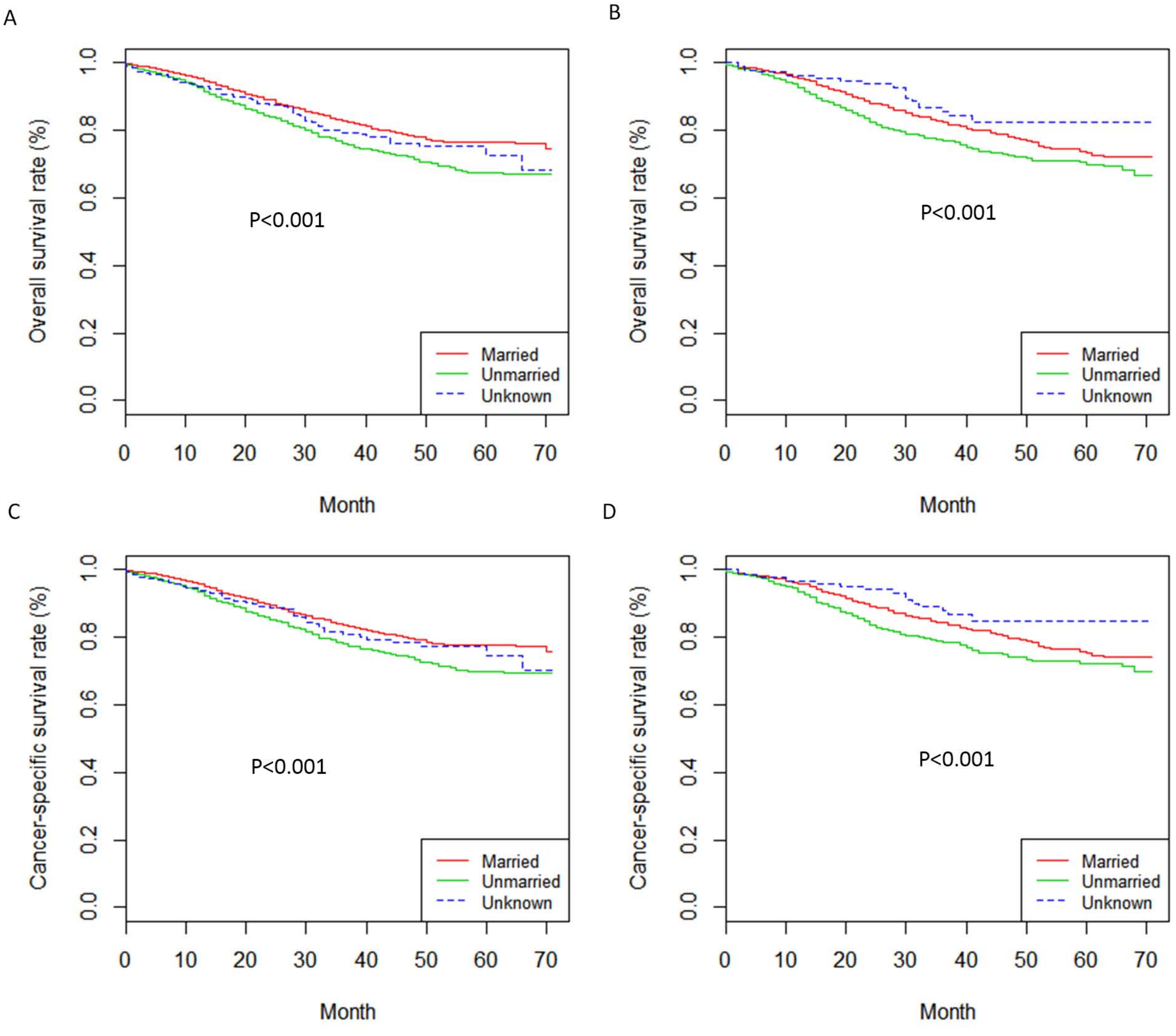
A
Overall survival rate (%)
0.0
0.2
0.4
0.6
0.8
1.0
P<0.001
Married
Unmarried
Unknown
0
10
20
30
40
50
60
70
Month
B
Overall survival rate (%)
0.0
0.2
0.4
0.6
0.8
1.0
P<0.001
Married
Unmarried
Unknown
0
10
20
30
40
50
60
70
Month
C
Cancer-specific survival rate (%)
0.0
0.2
0.4
0.6
0.8
1.0
P<0.001
Married
Unmarried
Unknown
0
10
20
30
40
50
60
70
Month
D
Cancer-specific survival rate (%)
0.0
0.2
0.4
0.6
0.8
1.0
P<0.001
Married
Unmarried
Unknown
0
10
20
30
40
50
60
70
Month

Supplementary table 1. Clinicopathological characterization of early-onset colon cancer patients stratified by marital status.

Variables	Married n (%)	Unmarried n (%)	Unknown n (%)	*P*
Total	**5862（52.2）**	**4679（41.7）**	**679（6.1）**	
**Sex**				**0.521**
Male	**2872（49.0）**	**2336（49.9）**	**344（50.7）**	
Female	**2990 (51.0)**	**2343 (50.1)**	**335 (49.3)**	
**Age**				**<0.001**
<30	**202 (3.4)**	**799 (17.1)**	**56 (8.2)**	
30-40	**1256 (21.4)**	**992 (21.2)**	**135 (19.9)**	
>40	**4404 (75.1)**	**2888 (61.7)**	**488 (71.9)**	
**Race**				**<0.001**
White	**4472 (76.3)**	**3287 (70.3)**	**447 (65.8)**	
Black	**657 (11.2)**	**1001 (21.4)**	**118 (17.4)**	
Other	**688 (11.7)**	**346 (7.4)**	**67 (9.9)**	
Unknown	**45 (0.8)**	**45 (1.0)**	**47 (6.9)**	
**Grade**				**<0.001**
I	**708 (12.1)**	**773 (16.5)**	**114 (16.8)**	
II	**3644 (62.2)**	**2635 (56.3)**	**379 (55.8)**	
III	**947 (16.2)**	**706 (15.1)**	**67 (9.9)**	
IV	**198 (3.4)**	**190 (4.1)**	**31 (4.6)**	
unknown	**365 (6.2)**	**375 (8.0)**	**88 (13.0)**	
**Site**				**<0.001**
Appendix	**686 (11.7)**	**856 (18.3)**	**103 (15.2)**	
Ascending colon	**720 (12.3)**	**602 (12.9)**	**68 (10.0)**	
Cecum	**812 (13.9)**	**656 (14.0)**	**84 (12.4)**	
Descending colon	**499 (8.5)**	**364 (7.8)**	**62 (9.1)**	
Hepatic flexure	**203 (3.5)**	**143 (3.1)**	**18 (2.7)**	
Large intestine, NOS	**105 (1.8)**	**82 (1.8)**	**12 (1.8)**	
Sigmoid colon	**2228 (38.0)**	**1458 (31.2)**	**273 (40.2)**	
Splenic flexure	**188 (3.2)**	**151 (3.2)**	**20 (2.9)**	
Transverse colon	**421 (7.2)**	**367 (7.8)**	**39 (5.7)**	
**AJCC.stage**				**<0.001**
I	**1316 (22.4)**	**1199 (25.6)**	**246 (36.2)**	
II	**1438 (24.5)**	**1088 (23.3)**	**155 (22.8)**	
III	**1917 (32.7)**	**1452 (31.0)**	**178 (26.2)**	
IV	**1191 (20.3)**	**940 (20.1)**	**100 (14.7)**	
**AJCC.T**				**<0.001**
T1	**1070 (18.3)**	**1056 (22.6)**	**220 (32.4)**	
T2	**577 (9.8)**	**383 (8.2)**	**56 (8.2)**	
T3	**2907 (49.6)**	**2135 (45.6)**	**271 (39.9)**	
T4	**1308 (22.3)**	**1105 (23.6)**	**132 (19.4)**	
**AJCC.N**				**<0.001**

N0	**2988 (51.0)**	**2482 (53.0)**	**421 (62.0)**	
N1	**1647 (28.1)**	**1212 (25.9)**	**142 (20.9)**	
N2	**1227 (20.9)**	**985 (21.1)**	**116 (17.1)**	
**AJCC.M**				**0.002**
M0	**4671(79.7)**	**3739(79.9)**	**579(85.3)**	
M1	**1191 (20.3)**	**940 (20.1)**	**100 (14.7)**	
**Tumor.size**				**<0.001**
<=2cm	**840 (15.7)**	**909 (21.1)**	**163 (28.5)**	
>2 to <= 5 cm	**2501 (46.7)**	**1649 (38.2)**	**193 (33.8)**	
>5 to <= 10 cm	**1757 (32.8)**	**1503 (34.9)**	**187 (32.7)**	
> 10 cm	**255 (4.8)**	**251 (5.8)**	**28 (4.9)**	
**SEER.stage**				**<0.001**
Localized	**2018 (34.4)**	**1718 (36.7)**	**318 (46.8)**	
Regional	**2571 (43.9)**	**1948 (41.6)**	**245 (36.1)**	
Distant	**1273 (21.7)**	**1013 (21.6)**	**116 (17.1)**	
